# Conceptualization and teaching health advocacy in undergraduate medical education: a document analysis

**DOI:** 10.1186/s12909-024-06039-0

**Published:** 2024-09-28

**Authors:** Femke E. de Bok, Jessie Hermans, Robbert J. Duvivier, Djoeke Wolff, Sijmen. A. Reijneveld

**Affiliations:** 1 Department of Health Sciences, University Medical Center Groningen, University of Groningen, Groningen, The Netherlands; 2 Parnassia Psychiatric Institute, The Hague, The Netherlands

**Keywords:** Health advocacy, Undergraduate medical education, Competency-based medical education, Document analysis, Social responsibility of physicians

## Abstract

**Background:**

Health advocacy is considered to be a core competence for physicians, but it remains unclear how the health advocacy role, despite being described in overarching competency frameworks, is operationalized in undergraduate medical education (UME). This study aimed to identify how health advocacy is conceptualized and taught in undergraduate medical curricula.

**Methods:**

We performed a qualitative analysis of curriculum documents from all eight medical schools in the Netherlands, all of which offered competency-based UME. Thematic analysis was used to code all the documents and generate themes on health advocacy conceptualization and teaching. To categorize the emerging themes, we used the framework of Van Melle et al. for evaluating the implementation of competency-based medical educational programs.

**Results:**

Health advocacy was mostly conceptualized in mission statements about social responsibility of future physicians, related to prevention and promoting health. We found key concepts of health advocacy to be taught mainly in public health and social medicine courses in the bachelor stage and in community-based clerkships in the master stage. Specific knowledge, skills and attitudes related to health advocacy were taught mostly in distinct longitudinal learning pathways in three curricula.

**Conclusion:**

Health advocacy is conceptualized mostly as related to social responsibility for future physicians. Its teaching is mostly embedded in public health and social medicine courses and community-based settings. A wider implementation is warranted, extending its teaching to the full width of medical teaching, with longitudinal learning pathways providing a promising route for more integrative health advocacy teaching.

## Background

Health advocacy is an important competence for today’s physicians to enable preventing disease and promoting health of individuals, communities, and populations [1]. The growing burden of chronic diseases in current society underlines the importance of health advocacy in daily practice and education of physicians [2]. Given their central role in the health care system, physicians can observe and address links between social, economic and political factors and health. They may use this position to improve health outcomes and reduce health inequities by promoting patient rights, engaging strategic partners and enhancing policy initiatives [3, 4]. Examples of such health advocacy at the population level include raising awareness of health inequities caused by poverty or campaigning against tobacco industries. Examples at the individual patient level include physicians referring a patient to a community health care service or ensuring that a patient has sufficient health information to navigate the health care system [5].

To ensure that physicians can act as effective health advocates, health advocacy has been included by medical societies as a core competence in several widely used competency frameworks for medical education [1, 6, 7]. These frameworks offer overarching outcome-based goals regarding the abilities required for professional competence in clinical practice [8], but the effective integration of health advocacy in competency based medical education (CBME) is considered challenging by educators and program directors in both undergraduate and postgraduate medical education [5, 9–12]. These challenges concern operationalizing the theoretical goals as described in competency frameworks into suitable curricular interventions for teaching health advocacy [5, 11–13]. Moreover, known barriers to curricular implementation comprise that the health advocacy role is perceived as less significant than other competencies by educators [14, 15] and that biomedical knowledge such as the pathophysiology of disease or pharmacological therapies, is emphasized over health advocacy topics in medical curricula [5].

The undergraduate stage of medical education is a pivotal period for introducing the concept of health advocacy to medical students [16]. Research suggests that by consolidating health advocacy training in undergraduate medical education (UME), students may acquire knowledge, skills and attitudes over the continuum of their formal training [16, 17]. Health advocacy training could contribute to developing students’ professional identity, making them better advocates for their patients, their communities and public health issues [13, 18]. Various authors have stressed the importance of formal health advocacy training in undergraduate medical curricula [4, 5, 17, 19], but uptake seems limited globally [20, 21]. A potential barrier might be that it remains unclear how the concept of health advocacy, despite being described in overarching competency frameworks, is operationalized in medical curricula. A better conceptualization of health advocacy may help to implement this competence, and similarly, may guide its practical teaching in curricula [5, 20, 21].

While prior studies have addressed how health advocacy is taught and assessed in postgraduate medical education (PGME), fewer studies have examined UME curricula [11, 12, 22]. Curriculum design is the process of defining and organizing elements of content, teaching and learning strategies, assessment and evaluation. The planned curriculum represents what is intended by designers [23]. Since health advocacy is underrepresented in UME and there seems to be a gap between the definition of the health advocacy competence in a framework and the operationalization of this competence in elements of curricular design, we aimed to explore how undergraduate medical schools conceptualize and teach the health advocacy competence in their planned curricula. This can provide more insight into curriculum development for health advocacy teaching.

## Methods

### Design and setting

We performed a qualitative document analysis on general curriculum documents from all eight medical schools providing undergraduate medical education in the Netherlands. The UME setting in the Netherlands provides a case in which a country offers competency-based medical education. All eight medical schools include a university teaching hospital and have curricula based on a national blueprint, the “Medical training framework 2020” [24]. This legally binding document defines all learning outcomes on the specific competencies that students need to master in order to become competent physicians, using the roles described by the Canadian Medical Education Directives for Specialists (CanMEDS). The CanMEDS framework is one of the most widely adopted frameworks for competency-based medical education and has “Health Advocate” as one of seven key physician roles [1].

### Sample and procedure

We set out to obtain a set of general curriculum documents per curriculum to obtain an overview of each curriculum. To retrieve these documents, curriculum program directors were approached between November 2021 and November 2022. After providing informed consent, we asked these program directors for several formal curriculum documents of their medical schools: general plans for each curriculum (“blueprints”), strategic documents and assessment plans and matrices. General descriptions of each curriculum and some assessment plans were also retrieved through publicly accessible websites. The documents obtained in this manner were used for the document analysis.

### Data handling and analysis

**Fig. 1:**
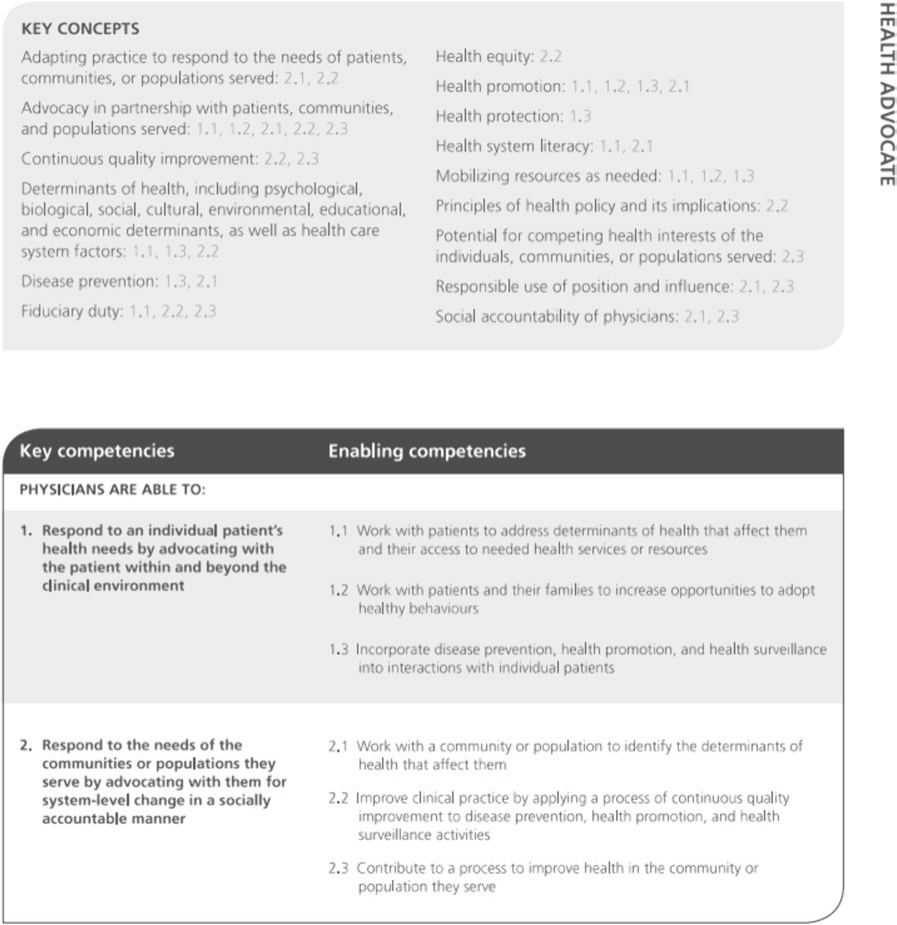
Key concepts, and key and enabling competencies of the CanMEDS role of health advocate

Regarding data handling, we used thematic analysis according to Bowen’s approach [25] to analyze the document dataset in Atlas.ti software. We did so by reading, finding and selecting fragments relevant for health advocacy in the curriculum documents. In this iterative process, FdB first read all the documents thoroughly and repeatedly to become familiar with the data. Second, text fragments relevant for health advocacy were inductively selected, using the description of the health advocate role of the CanMEDS framework [1]. The CanMEDS health advocate role comprises both key concepts and key and enabling competencies (see Fig. 1). These key elements of the health advocate role were used as guideline for thematic analysis. Third, FdB performed the initial coding process and category construction. JH and DW also coded a set of documents from two medical schools to cross-check the coding process. FdB then created a selection of deducted themes emerging from the coding process. The resulting selection of themes was discussed in the research team.

Regarding reporting analysis, we first described the sorts and nature of the documents as obtained. Next, we reported on the overarching themes that captured how health advocacy was conceptualized in the documents. Third, we reported on how health advocacy was taught, based on identified themes. These themes on health advocacy teaching were clustered using the organizing framework of Van Melle et al. [26] for evaluating implementation of competency-based medical educational programs. Van Melle et al. identified five core components of CBME, based on the construct of constructive alignment: outcome competencies, sequenced progression, tailored learning experiences, competency-focused instruction, and programmatic assessment. With these five core components and associated principle statements, they propose a common framework to guide the evaluation of CBME program implementation [26], which we used to sort the clustered codes and themes on how health advocacy was taught.

### Ethical issues

Ethical review board approval was received from The Dutch Association for Medical Education (NVMO, study number 2021.7.2).

## Results

### Description of curriculum documents

We obtained curriculum documents from the 2020–2021 academic year from all eight medical schools in the Netherlands. Each medical school provided both a medical bachelor’s and a medical master’s curriculum, resulting in 57 formal documents from a total of 16 bachelor’s and master’s programs to include in the analysis. Our dataset consisted of heterogeneous documents (see Table 1). In general, descriptions of the curriculum and the outline of the curriculum were provided on every medical school’s website. In strategic documents, mission and vision were described, as were didactic strategies for the curriculum. In curricular plans and course descriptions, information was found on blueprints of the curricula and the content of courses. The assessment plans included information about the assessment regulations and programmatic assessment.

**Table 1:** Description of types of curriculum documents as obtained for each medical school per stage of the curriculum, i.e., bachelor’s and master’s curriculum

	**Bachelor**	**Master**	
**Medical school A**	*General description curriculum* *Assessment plans*	*General description curriculum* *Strategic document* *Assessment plans*	
**Medical school B**	*General description curriculum* *Strategic document* *Curricular plan*	*General description curriculum* *Assessment plans*	
**Medical school C**	*General description curriculum* *Strategic document* *Curricular plan* *Course descriptions* *Assessment plans*	*General description curriculum* *Strategic document* *Curricular plan* *Course descriptions* *Assessment plans*	
**Medical school D**	*General description curriculum* *Curricular plan* *Course descriptions* *Assessment plans*	*General description curriculum* *Strategic document* *Curricular plan* *Course descriptions* *Assessment plans*	
**Medical school E**	*General description curriculum* *Strategic document* *Curricular plan* *Course descriptions* *Assessment plans*	*General description curriculum* *Strategic document* *Curricular plan* *Course descriptions* *Assessment plans*	
**Medical school F**	*General description curriculum* *Strategic document* *Assessment plans*	*General description curriculum* *Strategic document* *Assessment plans*	
**Medical school G**	*General description curriculum* *Assessment plans*	*General description curriculum* *Assessment plans*	
**Medical school H**	*General description curriculum* *Assessment plans*	*General description curriculum* *Strategic document* *Curricular plan* *Course descriptions* *Assessment plans*	
**Total number of documents**			***57***

### Conceptualization of health advocacy

We found health advocacy to be conceptualized as “social responsibility of students” in vision and mission statements of three medical schools. This was further specified as the need to prepare future physicians for their role in a changing society and health care environment, with a changing patient population (aging population with a growing proportion of comorbidities and chronic disease, diversity of population) and increasing health care costs. The concepts underlying social responsibility in these curricula were as follows: doctors being reactive to societal needs and doctors being committed to preventing disease, promoting health and contributing to improving the quality, efficiency and safety of health care. Two of these medical schools expressed the intention to focus medical education on prevention and health promotion and to shift from hospital-based education towards community-based settings in line with their mission of social responsibility.

### Teaching of health advocacy

Regarding ways of teaching health advocacy, we organized our findings according to the five clusters of the framework of Van Melle et al. [26]: outcome competencies, sequenced progressively, tailored learning experiences, competency-focused instruction and programmatic assessment.

#### Outcome competencies

In all the curricula, the intended curriculum outcomes for health advocacy were described as the CanMEDS health advocate role. In four curricula, the health advocacy competence was additionally clearly articulated in specific intended learning outcomes. Table 2 contains the identified learning outcomes in these curricula.

**Table 2:** Learning outcomes of health advocacy as described in four curricula, categorized into the two key competencies of the CanMEDS 2015 health advocate role

**Respond to an individual patient’s health needs by advocating with the patient within and beyond the clinical environment**
Incorporate disease prevention and health promotion into interactions with individual patients
Name levels of primary, secondary and tertiary prevention
Identify determinants of health that contribute to the experienced health of individual patients
Address access to needed care to health services or recourses for individual patients
Respond to individual patient’s needs, taking into account the context of patients
Integrate knowledge of determinants of health into medical practice
Apply methods of behavorial change and motivational interviewing on individual patient level
**Respond to the needs of the communities and populations they serve by advocating with them for system-level change in a socially accountable manner**
Form an opinion on societal issues in educational purpose settings
Insights and awareness of responsible use of position of physicians in society
Identify determinants of health that contribute to the experienced health of communities, populations and societal level
Apply knowledge of epidemiology on individual patient’s level and on population level
Insight in principles of health policy and its implications and the role of government and insurance companies on organization and financing of health care systems, costs and effectiveness of care
Insight in financing and regulation of health care systems
Insight in processes and interventions that can be used to improve health in communities or populations
Improve clinical practice by applying disease prevention and health promotion activities on population level
Analyze a public health problem from clinical practice
Contribute actively to improving quality, safety and efficacy of health care systems

#### Sequenced progressively

We could identify two didactic approaches for organizing the health advocacy competence in a developmental sequence: a longitudinal approach to learning and the use of entrustable professional activities (EPAs). First, three medical schools had a distinct learning pathway specifically focused on health advocacy, sometimes in combination with another competency such as collaboration. In these longitudinal learning pathways, learning experiences and teaching practices for health advocacy were designed to lead to progress and growth in competence and performance across the continuum of the bachelor’s or the master’s program. One of these medical schools provided a learning pathway for both the bachelor’s and the master’s programs. The developmental sequence in the bachelor’s program was described as knowledge of health advocacy and health systems in year 1, acquiring skills in simulated situations in year 2 and applying skills in practice in year 3. For the master’s program, the learning pathway was mentioned, but developmental sequence was not equally described as that in the bachelor's program. Another medical school provided a health advocacy learning pathway that was integrated in the semesters of the bachelor's program years 1, 2 and 3 by means of different assignments on key concepts of health advocacy.

The second didactic concept was the use of entrustable professional activities (EPAs) in two master’s curricula, described as specific tasks that integrate different competencies and must performed and assessed in the workplace during master’s phase clerkships. Health advocacy was an obligatory competence in four different EPAs regarding the following tasks: interprofessional collaboration, shared decision making, communication about diagnostic and therapeutic options and lifestyle counselling. In three EPAs, the health advocacy competence was described as obligatory, but the needed knowledge skills and attitudes were not articulated specifically in key concepts of health advocacy. In one curriculum, an EPA regarded counselling about healthy lifestyles and disease prevention. This specific EPA comprised knowledge of prevention, health promotion and lifestyle, and skills for motivational interviewing in consultation. The level students had to achieve to master this EPA, was defined as expected at supervised execution.

#### Tailored learning experiences

We identified learning experiences in social medicine courses in the bachelor’s curricula, in patient journey assignments and in distinct health advocacy learning pathways. Table 3 offers an overview of the tailored learning experiences found in the studied curricula. In courses dedicated to social medicine, we found key concepts of health advocacy such as prevention, public health aspects, global health, organization and efficiency of care. We also found examples of lectures involving the collaboration of different medical disciplines, i.e., lectures on the impact of disease on work and health by an occupational physician in a neurological course or lectures on prevention and lifestyle in a diabetes and obesity course. Another learning experience that included key concepts of health advocacy concerned patient journeys. These patient journeys involved following a patient for a period of several days during and after hospital admittance to the home environment. Health advocacy aspects consisted of interviewing these patients about social determinants of health and the perspective of the patient on health and recovery from disease. The overall goals of these assignments were to help students experience and investigate the impact of disease on patients lives. Learning experiences for health advocacy were also found to be part of the distinct health advocacy learning pathways in the in 3.3.2. section mentioned three curricula: two bachelor’s and one master’s programs.

**Table 3:** Health advocacy learning experiences and teaching practices

**Lectures** • Topics include public health, determinants of health, lifestyle interventions, organization of health care, prevention, health promotion
**Patient journey assignment: patient shadowing during and after hospital admittance, in the clinical environment and the home environment** • Interviewing patients, relatives and health care professionals to identify physical, social and psychological implications of disease • Write report with advice on how to improve health care processes that took place during patient journey
**Skill-building courses on motivational interviewing and health promotion** • Consultation training with other students or role-play based training • Consultation and communication about healthy behaviour
**Assignments on a specific population ** • Analysis of group of vulnerable patients, prepare interview with vulnerable patient • Analysis of employee with sickness absence, prepare interview with occupational medicine patient
**Scientific “PICO” with topic of lifestyle related disease, nutrition and epidemiology** • Searching strategies and write scientific report
**Community assignments** • Assignment with analysis of a living environment of patients • Group assignment on a specific societal problem or public health problem suggested by a community partner or societal institution, to come up with a plan to improve health care.

In the master’s curricula, key concepts of health advocacy were found mainly in community-based clerkships, especially social medicine rotations. Health advocacy was included in educational courses preceding and during clerkships on topics such as public health, the organization of health care, preventive child health care, work and healthcare and epidemiology. Teaching practices varied: lectures, workshops or courses on communication skills in social medicine and assignments for students to reflect on community-based practices. Most masters’ programs provided a community rotation varying from 6 to 12 weeks, during which students rotated between primary care, elderly care and social medicine.Three curricula described the development of longitudinal community clerkships to provide more educational continuity for competency development, especially for the health advocacy and collaboration competencies. Students experience day-to-day workplace activities in a community setting for at least 12 weeks where they are exposed to healthcare institutions focused on disease prevention instead of curation. Additionally, they work with communities and populations and collaborate in networks of care.

#### Competency-focused instruction

Didactic approaches to competency-focused instruction were found in the health advocacy learning pathways of three curricula, but were not described in further detail. This mostly regarded team-based learning, a teaching practice in which a coach or mentor offered individual reflection and feedback on the different assignments and learning experiences for health advocacy and other competencies, assisting students in their competency development and professional development.

#### Programmatic assessment

In all the curricula, we found the concept of programmatic assessment to be the basis for assessing competency development. In the bachelor’s curricula, assessment for health advocacy was specifically identified in the two curricula with advocacy learning pathways. Assessment instruments for health advocacy were described, but not in detail. The instruments that were described involved evaluating health advocacy assignments taught in the learning pathways and mentioning topics of health advocacy in written knowledge exams, such as public health topics and prevention*.*

In the master’s curricula, health advocacy assessment was mainly described as part of performance-based assessment during clinical and community clerkships. All CanMEDS competencies were assessed by a series of direct observations in the workplace environment using Mini‐Clinical Evaluation Exercises (mini-CEX) and 360° feedback from various assessors. We identified general descriptions of performance-based assessment of competencies, but without specification of the knowledge, skills and attitudes that were assessed for the health advocacy competence. In one master’s program, health advocacy assessment was described in more detail. This included an assessment of assignments related to study courses for community clerkships, an evaluation of knowledge in written exams and nine moments of workplace‐based assessment moments using mini-CEX and 360° feedback instruments.

## Discussion

### Main results

This study aimed to explore how health advocacy was conceptualized and taught in formal curriculum documents in a country with competency-based undergraduate medical curricula using an implementation framework. An overarching conceptualization of health advocacy was found in mission statements about social responsibility of future physicians, mostly related to the key concepts prevention and promoting health. Health advocacy teaching was mainly operationalized as learning experiences in public health and social medicine courses in the bachelor’s stage and in community-based and social medicine clerkships in the master’s stage. We found that more specific knowledge and skills for health advocacy were taught in distinct health advocacy learning pathways in a longitudinal approach, in both the bachelor’s and the master’s stages.

#### Interpretation of the main results

We found the concept of social responsibility in mission statements of three medical schools, reflecting an increasing awareness of the need to better prepare medical students for their role in society [13, 18, 27]. Social responsibility is defined as physicians remaining reactive to social demands and health needs, not only for their individualpatients but also for communities or the nation [27–29]. Health advocacy can provide a mean through which to operationalize social responsibility in medical curricula [27] and is thereby recognized as a competence that ought to be developed in medical students to foster socially responsible and socially accountable doctors [30–32]. Dharamsi et al stated that for a curriculum focused on developing social responsibility, medical students need to be part of a community practice and medical schools have to work together with the communities they serve to provide adequate advocacy opportunities [27]. Similarly, McDonald et al found that health advocacy often holds a prominent place in mandates and mission statements of various medical postgraduate curricula, but they also noted that this does not always lead to structural curricular interventions to teach health advocacy [12]. Our analysis demonstrated CanMEDS competences in the curriculum to be conceptualized either by examples of health advocacy via as social responsibility and community-based medicine or by a broader conceptualization.

According to our results, learning experiences related to health advocacy were found mainly in social medicine courses and clerkships as well as in community settings. These findings align with previous findings that health advocacy topics are mostly connected to public health and social medicine courses in undergraduate medical curricula [33–36]. Inherently, key concepts of health advocacy are closely related to topics found in public health or social medicine courses such as prevention, determinants of health, health promotion, health care delivery and population health management. Combined, they constitute the key components of the physician as health advocate [1, 5, 35]. Public health and social medicine-oriented components of medical education thus provide a logical opportunity to teach health advocacy. Different authors therefore state that in order to train physicians as health advocates, medical schools must commit more to implementing educational programs focused on public health topics and social medicine. Furthermore, they propose various frameworks and curriculum interventions to incorporate these topics into existing medical training instead of as isolated topics of health advocacy [36–39]. The curriculum documents used in our analysis involved mainly isolated social medicine courses and clerkships.

Our study showed that teaching health advocacy integrated with community activities mainly occurred in social medicine clerkships, and sometimes combined with family and elderly medicine clerkships. Several authors have tied the health advocacy role to social responsibility and to community-centered models of advocacy training [9, 40, 41]. This can be explained by several advantages of community-based teaching for health advocacy. First, by engaging in community learning activities, students develop a better acquisition of knowledge and skills for health advocacy, such as addressing social determinants of health and gaining insight into societal issues [40]. Second, actively involving students in community-based activities has a positive impact on students’ attitudes toward health advocacy as they become more aware of health disparities and more committed to practice in underserved communities [9, 33].  Finally, engaging with the local community outside the hospital setting, encourages medical students to draw connections between clinical presentations and social factors impacting health [9, 41]. It should be noted that in our data, this community-based teaching comprised a relatively short period of the curriculum: 6 to 12 weeks in the three-year master’s stage. We could not identify key concepts of health advocacy in the obligatory hospital-based clerkships that comprised on average a total of two years of mastery.

Health advocacy was mostly taught in regard to social medicine and community settings. Community-based teaching evidently has advantages for health advocacy teaching, but this restriction to these domains may provide limited exposure for students to health advocacy activities in clinical courses and clerkships. In the studied curricula, social medicine and public health courses and clerkships constituted only a minority of the six years of medical school. Hubinette et al [5] state that in order to train students as health advocates, health advocacy needs to be integrated into all aspects of undergraduate education. Health advocacy is a set of knowledge, skills and attitude that every physician should master and practice, not only in community medicine but also in clinical medicine. Therefore, we argue that health advocacy education should not be confined only to the domain of social medicine or public health but emphasis on health advocacy should also be placed on training in the clinical areas of undergraduate curricula.

We also found that health advocacy was included in other areas of the curricula, in ways separate from community-centered learning experiences, such as by means of various assignments in learning pathways aimed at health advocacy. Examples from our dataset used include the patient journey assignments, where key concepts of health advocacy as determinants of health and working with patients at the individual level both inside and outside the hospital environment were represented. Working with populations and key concepts of health policy and adapting practice to the need of populations were addressed in assignments about vulnerable populations and solving a community problem. The learning pathways started early in UME, following the course of the three year bachelor’s program, and in one medical school also the three years of master’s stage. According to our results, the learning pathways for health advocacy demonstrated an example of longitudinal curricular integration, but not all curricula in our analysis routinely used this didactical approach of sequenced progression. Several authors suggest that longer and earlier exposure to health advocacy opportunities, both community- and hospital-based, might be needed to better prepare students for independent practice. These authors also suggest that health advocacy is more than public health and social medicine alone and that it should be taught in a broader context, incorporating practice-based and skill acquisition elements of health advocacy into existing didactics. [4, 5, 16, 35, 36]. This finding aligns with what McDonald et al found in their recent reviews of health advocacy teaching in PGME: longitudinal curricula appear to be more fitting than short-term health advocacy projects or an isolated advocacy course, enabling students to engage with these concepts over the scope of months or years and throughout everyday learning activities [12]. By using a conceptual framework [26] for CBME implementation, we could infer that constructive alignment for the health advocacy competence was captured mostly in the curricula with longitudinal learning pathways.

#### Strengths and limitations

Our study has a number of strengths, the most important being that we included curriculum documents of all medical curricula of one country with a long tradition in competency-based medical education. For almost every curriculum, we included a quite complete set of information that we coded and categorized using an established framework [26]. By performing a qualitative analysis, we purposely coded our data in an inductive manner with cross-checking the coding of the data by other team members, to prevent bias and selectivity. Moreover, to our knowledge, few studies have examined how health advocacy teaching is positioned in documents about the intended curriculum in UME. Brender et al performed a document analysis of advocacy in U.S. medical schools where they found that advocacy was taught mainly in elective courses [42]. Other studies include  content analysis or scoping reviews of health advocacy curricula in postgraduate medical education [12, 22, 43].

We also note several limitations of our study. First, we had varying numbers of documents per curriculum and may have missed some curriculum documents. This may have led to incomplete assessments of some curricula, and thus underestimation somewhat the current teaching of health advocacy. Second, documents may not fully reflect the curriculum as is it delivered in practice, since curriculum documents represent the curriculum on paper, i.e., the intended curriculum [44]. The actual taught curriculum requires further study.

#### Implications for practice and research

In conclusion, we provided an overview of health advocacy conceptualization and teaching in intended curricula in a country with competency-based undergraduate medical education. Our findings have several implications for the practice of medical teaching and for further research.

First, the concept of social responsibility we found to be part of some mission statements, can provide insight for educators or curriculum leaders to be aware of the position of health advocacy teaching in medical curricula and to focus medical undergraduate education more on prevention and health promotion. Second, we showed that health advocacy education in social medicine courses provides promising learning experiences for acquiring knowledge, skills and attitudes for health advocacy. However, the literature suggests that a more integrative approach and earlier introduction of health advocacy may lead to better longitudinal development of this competence in UME. A wider implementation is warranted*, *extended to the full  witdth of medical teaching, including clinical areas of the curriculum. Longitudinal learning pathways provide a promising route for more integrative health advocacy teaching. Third, we found that an essential part of health advocacy teaching was in community-setting and through community-based activities. The advantages of this approach, which are supported by the literature, can also inform educators and increase their awareness of the inclusion and integration of community-based teaching of health advocacy as part of health advocacy education in their curriculum.

Finally, our analysis of curriculum documents provided valuable insight into which position health advocacy holds in curricula on paper as intended by curriculum designers. However, this is not the same as the curriculum in action, the delivered curriculum: the representation of how the intentions reflected in the curriculum on paper, appear in practice. Our findings can serve as a basis for further research on how health advocacy is taught in practice, to identify further promising routes for embedding health advocacy in medical teaching.

## Conclusions

By performing a qualitative analysis of curriculum documents, we provided an overview of health advocacy conceptualization and teaching in the intended curricula of undergraduate medical schools which offered competency-based UME. Health advocacy is conceptualized mostly as related to social responsibility for future physicians. Its teaching is mostly embedded in public health and social medicine courses and community-based settings. A wider implementation is warranted, extending its teaching to the full width of medical teaching, with longitudinal learning pathways providing a promising route for more integrative health advocacy teaching.

## Data Availability

The datasets used and/or analyzed during the current study are available from the corresponding author upon reasonable request.

## Abbreviations

CanMEDS: Canadian Medical Education Directives for Specialists
CBME: Competency Based Medical Education
EPAs: Entrustable Professional Activities
Mini-CEX: Mini‐Clinical Evaluation Exercises
PGME: Postgraduate Medical Education
UME: Undergraduate Medical Education

## Data availability

No datasets were generated or analysed during the current study.
